# Evaluation of match-running distances covered by soccer players during the UEFA EURO 2016

**DOI:** 10.17159/2078-516X/2019/v31i1a6127

**Published:** 2019-01-01

**Authors:** A Kubayi

**Affiliations:** Department of Sport, Rehabilitation and Dental Sciences, Faculty of Science, Tshwane University of Technology, Pretoria, South Africa

**Keywords:** movement patterns, high-speed running, training

## Abstract

**Background:**

Despite a substantial body of literature on match-running distances covered by soccer players in domestic leagues, there appears to be limited information on the Union of European Football Associations (UEFA) Euro competitions.

**Objective:**

The aim of this study was to analyse the match-running distances covered by soccer players during the UEFA Euro 2016.

**Methods:**

A multiple-camera tracking system (InStat Ltd) was used to analyse 228 observations of soccer players who played 15 full matches during the tournament. The outfield players were categorised according to the following playing positions: central defenders (CDs), n=58; wide defenders (WDs), n=45; central midfielders (CMs), n=53, wide midfielders (WMs), n=38; and attackers (ATs), n=34. Data were reported as means with 95% confidence intervals (CI). A one-way analysis of variance (ANOVA) was undertaken to examine the significant differences among players based on playing positions.

**Results:**

The results indicated that the overall total distance covered by players was 10 350 m, ranging from 8 446 m to 12 982 m. ATs covered the longest distance in high-speed running (872 m; 95% CI = 813–931), while CDs covered the shortest distance (542 m; 95% CI = 503–581). A statistically significant difference was observed in high-speed running among players (F (4 223) = 36.92, P=0.001).

**Conclusion:**

The findings of this study provide soccer scientists and coaches with important information to design and implement training sessions in order to elucidate the physical demands of players in view of successful team performance.

Soccer is the world’s most popular sport played on all continents.^[1–3]^ Its popularity coincides with the need for research into the match performance analysis in soccer. In recent years, technological advances have included the introduction of increasingly sophisticated motion analysis systems now being used in elite soccer.^[4]^ The use of these advanced approaches has made new methods more feasible and accessible, allowing for a greater volume of data to be collected in less time.^[5]^ To obtain physical performance indicators among soccer players, a variety of methods, such as visual evaluation, the Global Positioning System, and the semi-automated multi-camera tracking system have all been recommended in the literature.^[6]^

However, the most commonly used method is the semi-automated multi-camera tracking system^[7]^ which provides more precise information about the technical and physical performance indicators of the players during soccer matches. The validity of the semi-automated multi-camera tracking system has been demonstrated in several previous studies.^[8,9]^ More specifically, the semi-automated multi-camera tracking system describes the technical actions of players and allows the monitoring of the total running distances covered at various intensities.^[10]^ This system, which is based on numerous calibrated cameras, allows the monitoring of all players on the pitch during the match, both with or without the ball. Consequently, coaches and scientists can record the players’ technical, tactical and physical data simultaneously.^[11]^

For example, previous research^[12]^ has reported that soccer players generally cover a total distance of between 9 and 14 km during a match, with much of the game played at a low intensity, such as standing, walking and jogging. Specifically, Bradley et al.^[13]^ found that wide and central midfielders (WMs; CMs) covered longer distances than wide defenders (WDs), central defenders (CDs), and attackers (ATs). Likewise, WDs, CMs, and ATs covered greater distances in high-intensity running compared to CDs.^[13]^

Although extensive literature has analysed the movement patterns among professional players in domestic leagues,^[4,5,11]^ there is limited information on the UEFA European Championship. Therefore, a more detailed analysis of the movements displayed by soccer players in such a competition may provide a better understanding of the physical demands of the game.^[14]^ Thus the purpose of this study was to analyse the match-running distances covered by soccer players during the UEFA EURO 2016.

## Methods

### Match data and participants

A total of 228 soccer players (with the exception of the goalkeepers), who played 15 full matches during the UEFA EURO 2016 tournament were observed and their movements analysed. The outfield players were grouped according to one of five playing positions as follows: CDs (*n*=58), WDs (*n*=45), CMs (*n*=53), WMs (*n*=38), and ATs (*n*=34). To ensure confidentiality, match-running data were analysed without any reference to specific players and/or teams. Permission to conduct the study was granted by the InStat Company, and the study’s protocol was approved by the Faculty of Science Research Ethics Committee of the Tshwane University of Technology, Pretoria, South Africa.

### Data collection

The match-running distance of players was monitored using the multiple-camera tracking system (InStat). Physical data were captured with video cameras installed in pairs on each of the two main stands of a soccer stadium. The portable InStat fitness system set includes two video cameras positioned on tripods on one of the stands, with each of the two cameras covering half of the field. Previous research has reported the accuracy of the InStat tracking system in assessing physical performance.^[9]^ The physical performance movements of the soccer players were classified as follows: walking (0–7 km/h), jogging (7.1–14.5 km/h), running (14.6–20 km/h), high-speed running (20.1–25 km/h) and sprinting (>25 km/h).

### Statistical analysis

Data were reported as means with 95% confidence intervals (CI). A one-way analysis of variance (ANOVA) was used to assess the significant differences among players according to playing positions (i.e., CD, WD, CM, WM, and AT). Where the F-ratio was significant (P≤0.05), the Tukey HSD post-hoc test was undertaken for further analysis. Data were analysed by means of the Statistical Package for the Social Sciences (SPSS), version 25.0 (SPSS Inc., Chicago, IL, USA).

## Results

Table 1 presents distances covered during entire matches according to players’ field positions. It was found that an average total distance covered during the matches played was 10 350 m, ranging from 8 446 m to 12 982 m. Specifically, CMs (10 981 m; 95% CI = 10 787–11 174), ATs (10 532 m; 95% CI = 10 313–10 751), and WMs (10 534 m; 95% CI = 10 374–10 693) significantly covered a greater total distance compared to WDs (10 274 m; 95% CI = 10 097–10 451) and CDs (9 423 m; 95% CI = 9 300–9 546). The results indicated that CDs covered longer walking distances (3 634 m; 95% CI = 3 559–3 710) than players in other outfield positions. There was a statistically significant difference in walking distances for the groups of players (F (4 223) = 8.52, P=0.001). Post-hoc comparisons using the Tukey HSD post-hoc test showed that the walking distance for CDs was significantly (P<0.05) different from that of CMs (3296 m; 95% CI = 3 208–3 383). WDs (3 493 m; 95% CI = 3 394–3 592), WMs (3 520 m; 95% CI = 3 426–3 614), and ATs (3532 m; 95% CI = 3 414–3 651) did not differ significantly from either CDs or CMs.

With regard to high-intensity running, ATs covered the longest distance in high-speed running (872 m; 95% CI = 813–931), while CDs covered the shortest distance (542 m; 95% CI = 503–581). A statistically significant difference was observed in high-speed running between players (F (4 223) = 36.92, P=0.001). The Tukey HSD post-hoc analyses indicated that the high-speed running mean score for ATs was significantly (P<0.05) different from that of CDs. However, the high-speed running for ATs and CDs did not differ significantly from those of WDs (799 m; 95% CI = 751–848), CMs (857 m; 95% CI = 801–914), and WMs (856 m; 95% CI = 813–900).

## Discussion

The results of this study indicated that the average total distance covered by players was 10 350 m throughout the matches analysed. These findings are slightly lower than those of Di Salvo, et al.,^[14]^ who found that 300 players in the Spanish Premier League (La Liga) and UEFA Champions League matches covered an average of 11 393 m. The discrepancy between the present findings and those of previous studies probably demonstrates that in modern soccer it is not about how much distance a player covers but knowing where to go at the right time and how to use space efficiently during a match.

When the data were analysed according to playing position, midfielders covered greater distances compared to defenders and forwards. This finding could be explained by the fact that midfielders play an important role as a link between defenders and attackers ^[14]^ which requires them to shuttle between the attack and defence. Previous studies have provided empirical evidence showing that midfield players had higher VO_2max_ values than fullbacks and forwards ^[14],^ thus emphasising their indefatigable role associated with long-distance coverage during a match.

Another important finding in this study was that WDs covered more distance overall than their CD counterparts. A plausible reason for this result could be that in modern soccer WDs are encouraged to advance to the final third of the field in order to execute crosses into the opposition goal area. This notion is supported by the research of Di Salvo, et al. ^[15]^ who indicated that fullbacks are required to operate in both attacking and defensive contexts in modern match tactics. The finding in which CMs covered greater total distances than other players (i.e. CDs, WDs, WMs, and ATs) is noteworthy. Although it was not ascertained in the current study, the tactical duties of CMs could be different from those of other playing positions. The current observations may suggest that CMs are subject to greater overall exertion requiring higher fitness levels. The longer distances covered by CMs may be associated with team formations and/or playing styles employed by European teams. ^[5,10,15]^

With regard to distances covered by players at different work intensities, CDs covered greater total distances in low-intensity activities, such as walking. It has been established that CDs spend most of time during the game engaged in low-intensity activities without the ball, probably as a result of their involvement being limited to defensive actions. ^[15]^ The results also showed that ATs covered greater distances of high-speed running compared to players in other positions. The modern approach of attacking players applying higher pressure on the opposition by becoming the first defensive line may also contribute to this finding. The ability to accelerate and reach maximal speeds quickly is an important component of game-deciding situations in soccer. ^[13]^ A requirement for such movement patterns by attacking players in this study can be attributed to the need to complete fast movements away from defending players to create space or capitalise on goal-scoring opportunities. ^[15]^

Although this study provided novel information on the distances covered by European players, a few limitations should be taken into account. First, due to the various categorisations and technologies employed, caution is necessary in the interpretation of the present findings and comparing them with those reported in the literature. ^[14]^ Second, the analysis of a small number of matches during this competition also limits the generalisation of the current findings. Therefore, future studies involving larger samples of European tournaments, especially UEFA EURO Championships, are needed to clarify the findings of the present study.

## Conclusion

The current findings showed that midfielders covered greater total distances than defenders and forwards. While CDs spent most time during the game-walking and jogging, ATs covered greater distances in high-intensity activities, such as high-speed running, compared to other positions. The results could provide valuable information for soccer scientists and coaches in planning and implementing position-specific training programmes, thereby maximising team performance.

## Figures and Tables

**Table 1:** Average match-running distances covered during UEFA EURO 2016

	95% CI				

	N	Mean	Lower bound	Upper bound	p-value
	
**Total distance (m)**					0.001*
**CD**	58	9423	9300	9546	
**WD**	45	10274	10097	10451	
**CM**	53	10981	10787	11174	
**WM**	38	10534	10374	10693	
**AT**	34	10532	10313	10751	
**Walking distance (m)**					0.001*
**CD**	58	3634^a^	3559	3710	
**WD**	45	3493	3394	3592	
**CM**	53	3296	3208	3383	
**WM**	38	3520	3426	3614	
**AT**	34	3532	3414	3651	
**Jogging distance (m)**					0.001*
**CD**	58	3882	3782	3983	
**WD**	45	4159	3998	4320	
**CM**	53	4717	4585	4850	
**WM**	38	4196	4035	4358	
**AT**	34	4274	4057	4491	
**Running distance (m)**					0.001*
**CD**	58	1285	1202	1369	
**WD**	45	1627	1506	1748	
**CM**	53	2000	1863	2136	
**WM**	38	1780	1678	1882	
**AT**	34	1672	1561	1782	
**High-speed running (m)**					0.001*
**CD**	58	542	503	581	
**WD**	45	799	751	848	
**CM**	53	857	801	914	
**WM**	38	856	813	900	
**AT**	34	872^b^	813	931	
**Sprinting distance (m)**					0.001*
**CD**	58	80	70	91	
**WD**	45	195	171	220	
**CM**	53	112	95	130	
**WM**	38	183	157	208	
**AT**	34	186	159	213	

* Statistically significant at P<0.05;

a Significantly different from CM, P< 0.05;

b Significantly different from CD, P<0.05.

CD, Central defender; WD, Wide defender; CM, Central Midfielder; WM, Wide Midfielder; AT, Attacker; N, Number of participants; CI, Confidence interval
